# Electrospun Magnetic Ionic Liquid Based Electroactive Materials for Tissue Engineering Applications

**DOI:** 10.3390/nano12173072

**Published:** 2022-09-04

**Authors:** Liliana C. Fernandes, Rafaela M. Meira, Daniela M. Correia, Clarisse Ribeiro, Eduardo Fernandez, Carmen R. Tubio, Senentxu Lanceros-Méndez

**Affiliations:** 1 Physics Centre of Minho and Porto Universities (CF-UM-UP), University of Minho, 4710-057 Braga, Portugal; 2 LaPMET—Laboratory of Physics for Materials and Emergent Technologies, University of Minho, 4710-057 Braga, Portugal; 3 IB-S—Institute of Science and Innovation for Sustainability, University of Minho, 4710-057 Braga, Portugal; 4 Centre of Chemistry, University of Minho, 4710-057 Braga, Portugal; 5 BCMaterials, Basque Center for Materials, Applications and Nanostructures, UPV/EHU Science Park, 48940 Leioa, Spain; 6 IKERBASQUE, Basque Foundation for Science, 48013 Bilbao, Spain

**Keywords:** electrospun fibers, electroactive materials, piezoelectric polymers, ionic liquids, tissue engineering

## Abstract

Functional electrospun fibers incorporating ionic liquids (ILs) present a novel approach in the development of active microenviroments due to their ability to respond to external magnetic fields without the addition of magnetic particles. In this context, this work reports on the development of magnetically responsive magneto-ionic fibers based on the electroactive polymer poly(vinylidene fluoride) and the magnetic IL (MIL), bis(1-butyl-3-methylimidazolium) tetrathiocyanatocobaltate ([Bmim]_2_[(SCN)_4_Co]). The PVDF/MIL electrospun fibers were prepared incorporating 5, 10 and 15 wt.% of the MIL, showing that the inclusion of the MIL increases the polar *β*-phase content of the polymer from 79% to 94% and decreases the crystallinity of the fibers from 47% to 36%. Furthermore, the thermal stability of the fibers decreases with the incorporation of the MIL. The magnetization of the PVDF/MIL composite fibers is proportional to the MIL content and decreases with temperature. Finally, cytotoxicity assays show a decrease in cell viability with increasing the MIL content.

## 1. Introduction

The combination of cells, bioactive factors and scaffolds for biomedical applications, including tissue engineering (TE), allows the development of biological substitutes to restore and/or improve tissue functions [1]. Scaffolds represent a key element on TE approaches, providing the structural support for cell growth as well as the necessary stimuli for proper tissue regeneration [2,3]. In this sense, active smart materials are able to reversibly modify one or more of their functional or structural properties in a controlled and reproductible fashion when subjected to external stimuli, such as temperature, stress, electric or magnetic fields, among others. [4,5]. The implementation of those active materials is able to mimic the dynamic natural cell environment by providing mechanic and/or electric stimuli similar to the ones present in the human body [6].

Among smart materials, electroactive materials have shown suitability for TE applications due to their ability to convert a mechanical deformation into an electrical potential and vice-versa, without need of an external voltage source [5,7]. Thus, smart materials are of great interest for recreating functional biomimetic microenvironments. This field is taking advantage of piezoelectric materials to mimic electrical and mechanical cues existing in the living tissues, and thus assist in their natural regeneration [8].

Poly(vinylidene fluoride) (PVDF) is the most investigated and used piezoelectric polymer due to its large piezoelectric response [9]. Together with being biocompatible and biostable, PVDF shows high mechanical strength, thermal stability and chemical resistance [10,11]. Moreover, this semicrystalline polymers can be synthetized in a variety of shapes, including films, fibers, membranes and spheres [12,13], and it can also morphologically mimic specific tissue microenvironments. PVDF exhibits an unusual polymorphism, showing four main crystalline phases (*α*, *β*, *γ* and *δ*). The *β*-phase is responsible for the largest piezoelectric response and is, consequently, the most desirable for TE applications [14]. Generally, the piezoelectric *β*-phase is achieved by mechanical stretching of the *α*-phase, poling under high electrical fields [10] or when specific fillers are incorporated, such as barium titanate particles [15], cobalt ferrites [16], zeolites [17] and ionic liquids [18,19].

Ionic liquids (ILs) are a diversified group of salts composed of organic cations and a variety of anions that present a melting temperature typically below 100 °C [20]. They are under increasing attention due to their outstanding properties, including negligible vapor pressure [21], high ionic conductivity [22], electrochemical and thermal stability [23], which make them suitable for the development of novel multifunctional materials with tailorable functionality and a variety of processing possibilities, as well as compatible with additive manufacturing technologies [24]. Those ionic smart materials are finding applications in areas such as sensors and actuators, batteries, catalysis and TE applications, among others [25].

Up to now, few studies have reported both the development and application of polymer based-ILs as scaffolds for biomedical applications, most of them being focused on the development of electromechanical actuators [26]. It has been shown that 1-ethyl-3-methylimidazolium bis(trifluoromethylsulfonyl)imide [C_2_mim][NTf_2_] incorporation into the PVDF polymeric matrix modifies the polymer crystallization process, induces the PVDF crystallization into the *β*-phase and also increases the electrical conductivity of the composite materials [27]. Moreover, the incorporation of [C_2_mim][NTf_2_] decreases the Young’s modulus. Similar results were obtained in [28]. Regardless of IL content and type, it has been observed that the bending response can be tuned by the selection of anion and cation sizes.

Aiming to develop suitable platforms for muscle tissue regeneration, biocompatible IL/PVDF composite films [24] were developed comprising two different ILs: 1-butyl-3-methylimidazolium chloride [Bmim][Cl] and 2-hydroxyethyl-trimethylammonium dihydrogen phosphate [Ch][DHP]. An increase in the PVDF electroactive *β*-phase content and the degree of crystallinity is obtained with the presence of both ILs due to the strong ion-dipole interactions, indicating also that the ILs act as a nucleating agent for polymer crystallization. Besides enhancing the electrical conductivity, the incorporation of the ILs also influences the thermal properties of the films. Regardless of the IL type, a decrease in the Young´s modulus is observed and the ILs act as a PVDF plasticizer. Cell culture assays showed that cells adhered and proliferated in all samples, the myoblast cells showing a compact cytoskeleton particularly when growing on [Bmim][Cl]/PVDF. In another study, [C_2_mim][NTf_2_] was incorporated into the PVDF fibers [29] to fabricate ionic electroactive fibers for TE applications. It was demonstrated that the [C_2_mim][NTf_2_]/PVDF fiber mats are not cytotoxic and enhanced C2C12 cell viability.

PVDF-based electrospun membrane composites have been developed for biomedical applications, including tissue regeneration [30], incorporating different contents (5, 10 and 15 wt.%) of iron oxide nanoparticles (Fe_3_O_4_) or a biocompatible IL (choline bis(trifluoromethylsulfonyl) imide ([Ch][TFSI]. While the morphology and degradation temperature remain similar to the addition of both fillers, an increase in the *β*-phase was observed with the different fillers added together, with a small increase in the thickness of the IL containing fibers. Additionally, good incorporating efficiencies (>93%) were observed for the Fe_3_O_4_ nanoparticles. All composite fibers showed high compatibility to L929 fibroblasts [30]. Additionally, PVDF and PHBV-based fibers and films were also processed using the IL choline acetate ([Ch][Ac]) as a filler [31]. The IL addition decreases the fibers’ orientation, thermal stability and surface wettability, and increases the films’ surface roughness of the composites and the ionic conductivity. Up to a concentration of 10 wt.% of the IL in either morphology, compatibility with C2C12 myoblasts is observed.

Besides the strong interest devoted to the development of IL-based composites for TE, no studies have been performed on the development of composites combining piezoelectric polymers with magnetic ionic liquids (MILs) for TE applications. These types of ILs exhibit a magnetoelectric response, i.e., the development of an electric potential variation under applied magnetic field, associated with a magneto-ionic response [32,33]. Thus, the application of an external magnetic field will allow the development of an electrically varying microenvironment to the cultured cells [25] without the use of magnetic nanoparticles.

In this sense, the main focus of the present work is the development of magneto-ionic electrospun composite fibers based on PVDF and bis(1-butyl-3-methylimidazolium) tetrathiocyanatocobaltate [Bmim]_2_[(SCN)_4_Co] comprising different MIL contents (5, 10 and 15 wt.%), the aim being to explore their processability and potential for tissue engineering applications. Their morphological, physical, chemical and magnetic properties were evaluated, together with their cytotoxic response. The physical-chemical characterization, magnetic response and biocompatibility represent a complete set of data to demonstrate the potential of the materials to be explored for tissue engineering applications. The techniques are used to properly understand the influence of the inclusion of the IL into the PVDF matrix, namely in the polymer crystallization in the polar *β*-phase, the most relevant phase for biomedical applications. Additionally, the magnetic properties have been evaluated for potential cell stimulation under dynamic magnetic conditions. Finally, the cytotoxicity of the materials has been addressed, determining the maximum IL concentration to be incorporated in the polymer matrix without inducing a toxic cell response.

## 2. Materials and Methods

### 2.1. Materials

The polymer PVDF (5130) and the solvent N,N-dimethylformamide (DMF, 99.5%) were purchased from Solvay (Carnaxide, Portugal) and Merck (Darmstadt, Germany) respectively. The MIL [Bmim]_2_[(SCN)_4_Co] (purity > 99%) was acquired from Iolitec (Heilbronn, Germany).

### 2.2. Sample Preparation

PVDF and PVDF/[Bmim]_2_[(SCN)_4_Co] electrospun fibers were processed as follows: Briefly, a solution of PVDF/DMF with a proportion of 15/85 wt.% was prepared under magnetic stirring at room temperature until complete dissolution of the polymer. For the composite fibers, different MIL contents (5, 10 and 15 wt.%) were dispersed in DMF under mechanic stirring at room temperature and PVDF was added until complete dissolution. Then, the polymer solution was placed in a 10 mL plastic syringe fitted with a steel needle (inner diameter of 0.5 mm). After processing parameter optimization, the electrospinning process was conducted at 14 kV with a high voltage power supply from Glassman (PS/FC30P04 model) with a solution feed rate of 0.4 mL h^−1^ applied with a syringe pump (from Syringepump). PVDF and PVDF/[Bmim]_2_[(SCN)_4_Co] fibers were collected in a rotating aluminum substrate (1500× per min) in order to obtain an aligned fiber morphology. The processing parameters are summarized in Table S1.

### 2.3. Samples Characterization

The morphology of PVDF and PVDF/[Bmim]_2_[(SCN)_4_Co] electrospun fibers with different MIL contents were analyzed by scanning electron microscopy (SEM, NanoSEM–FEI Nova 200) with an accelerating voltage of 10 kV. Before the analysis, a thin gold layer was coated in the samples using a sputter coating (Polaron, model SC502). The fiber diameter distribution was calculated to be over 50 fibers with the Image J software (Image J) from the SEM images.

Fourier Transform Infrared (FTIR) measurements in the Attenuated Total Reflectance (ATR) mode was used to evaluate the electroactive *β*-phase of the polymer. Measurements were performed with a Jasco FT/IR-4100 apparatus at room temperature scanning from 4000 to 600 cm^−1^ using 64 scans at a resolution of 4 cm^−1^. The electroactive *β*-phase fraction *F*(*β*) was evaluated using Equation (1) [34].

(1) F(β)=Aβ(KβKα)(Aα+Aβ)

where *A_α_* is assigned to the absorbance at 766 cm^−1^ attributed to the PVDF *α*-phase, and *A_β_* represents the absorbance at 840 cm^−1^ attributed to the PVDF *β*-phase. *K_α_* and *K_β_* are the corresponding absorption coefficients: 6.1 × 10^4^ and 7.7 × 10^4^ cm^2^·mol^−1^.

Thermogravimetric analysis (TGA) was carried out in N_2_ atmosphere over a temperature range from 40 to 800 °C using a Mettler Toledo TGA/SDTA 851e system. The samples were heated at a rate of 10 °C·min^−1^.

Differential scanning calorimetry (DSC) experiments were conducted at a heating rate of 10 °C·min^−1^ in the temperature range from 25 to 200 °C under N_2_ atmosphere with a Mettler Toledo DSC 822e equipment. Measurements were carried. Determination of the degree of crystallinity (*X_c_*) was carried out after Equation (2) [34]:

(2) $$X_{c}=\frac{\Delta H}{x\Delta H_{\alpha }+y\Delta H_{\beta }}$$

where Δ*H* is the melting enthalpy of PVDF, Δ*H_α_* and Δ*H_β_* are the melting enthalpies of the *α* (93.07 J·g^−1^) and *β* phases (103.4 J·g^−1^) of PVDF, respectively, and *x* and *y* are the *α* and *β* phase content in each sample [34], respectively, obtained from the FTIR results and Equation (1).

The zero-field cooling magnetization curves of the electrospun fibers were performed at 1 kOe with a temperature rate of 10 K·min^−1^ using a superconducting quantum interference device (SQUID, Quantum Designs MPMS 3). All fibers were cut with ceramic tweezers to avoid contamination and placed on quartz sample holders fixed with teflon tape to avoid losing the sample while measuring on the VSM. On the other hand, the ionic liquid hysteresis loop was measured using a polymeric capsule inserted on a quartz tube. Prior to the measurement, a hysteresis loop at room temperature was obtained for each sample up to 20 kOe. The magnetic hysteresis loops up to 10 kOe at different temperatures were measured using a EZ7 MicroSense vibrating sample magnetometer (VSM).

For in vitro assays, circular samples from PVDF fibers comprising different MIL concentrations were cut into 13 mm diameter circles. For the sterilization process, the samples were placed in a 24-well cell culture plate and exposed to ultraviolet light (UV) for 2 h (1 h each side). Then, they were washed five times with a sterile phosphate buffer saline (PBS) solution for 5 min each time. L929 cells were cultivated in Dulbecco´s Eagle´s medium (DMEM, Gibco) containing 4.5 g·L^−1^ glucose, 10% fetal bovine serum (FBS, Biochrom) and 1% penicillin/streptomycin (P/S, Biochrom) under standard culture conditions (37 °C in a 95% humidified air containing 5% CO_2_). The culture medium was changed every 3 days and the cells were collected when ~70% confluence was reached. The cytotoxicity of the PVDF/[Bmim]_2_[(SCN)_4_Co] fibers were evaluated by 3-(4,5 dimethylthiazol-2-yl)-5-(3-carboxymethoxyphenyl)-2-(4-sulfophenyl)-2H-tetrazolium (MTS, Promega) assay. After the sterilization process, the samples were exposed for 24 h to culture medium at 37 °C in a 95% humidified air containing 5% CO_2_. In parallel, cells were seeded in 24-well tissue culture polystyrene plates for 24 h to allow cell attachment. After 24 h, the culture medium of seeded cells was removed and the culture medium that was in contact with the different samples was added. Subsequently, the cells were incubated for 72 h and their metabolic activity was quantified by an MTS assay. For this, the medium of each well was replaced by fresh medium containing MTS solution (dilution of 1:5 in DMEM) and incubated at 37 °C in a 5% CO_2_ incubator. The absorbance was measured, using a microplate reader, at 490 nm, after 2 h of incubation. Four replicate absorbance measurements were carried out at 490 nm using a microplate reader. The metabolic activity, as an indicator of cell viability and cytotoxicity, was calculated according to Equation (3).

(3) $$Metabolic\;activity\;\left(\%\right)=\frac{absorbance\;of\;sample\;}{absorbance\;of\;negative\;control\;}\times \;100$$

## 3. Results

### 3.1. Morphology of the Fibers 

**Figure 1:**
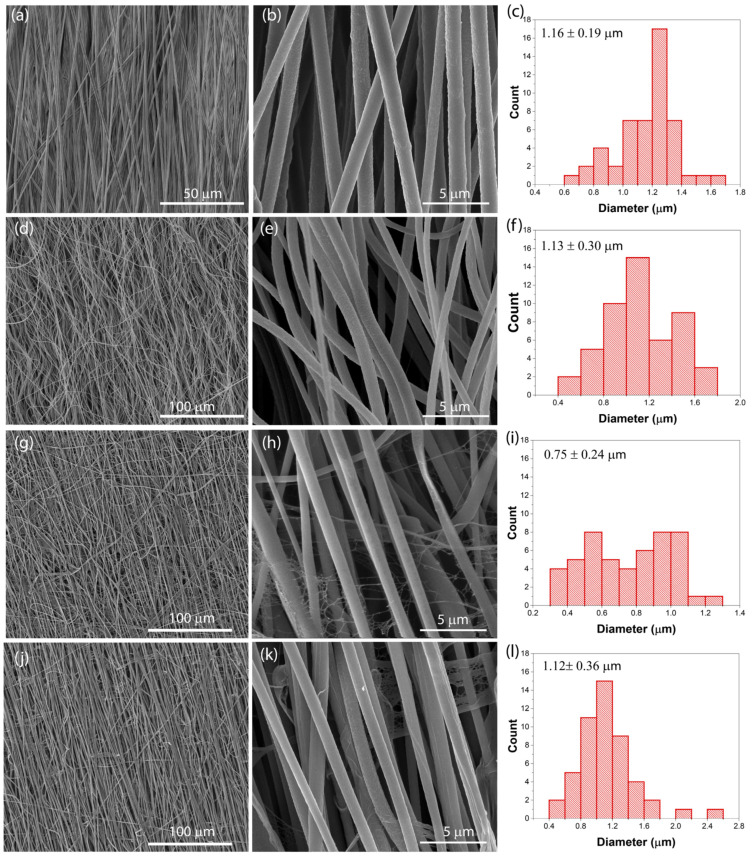
Representative SEM images of the PVDF composite fibers containing (**a**,**b**) 0, (**d**,**e**) 5, (**g**,**h**) 10 and (**j**,**k**) 15 wt.% of [Bmim]_2_[(SCN)_4_Co] and the corresponding average fiber diameter distribution (**c**,**f**,**i**,**l**), respectively.

The morphology of the developed electrospun fibers was evaluated by SEM. Figure 1 shows representative SEM images and the average fiber diameter distribution of the oriented neat PVDF and PVDF/[Bmim]_2_[(SCN)_4_Co] fibers with different MIL contents. 

For the PVDF fibers, oriented fibers are observed, compatible with the collection of the fibers in a rotating drum [35], presenting a smooth and flat surface (Figure 1a,b) with an average fiber diameter of 1.16 ± 0.19 µm (Figure 1c). The inclusion of the MIL [Bmim]_2_[(SCN)_4_Co] into the PVDF matrix does not promote changes in the surface morphology of the fibers, remaining with a smooth surface without the presence of beads. Upon the inclusion of 5 wt.% MIL into the PVDF polymer matrix (Figure 1d,e) a smooth surface is observed with no variations in the fiber diameter of the sample to 1.19 ± 0.23 µm (Figure 1f) with respect to the pristine polymer. With increasing the MIL content up to 10 wt.%, a decrease in the fiber diameter is observed to 0.75 ± 0.22 µm (Figure 1j) as a result of the number of thin fibers that occur with the MIL incorporation, mainly attributed to the electrical conductivity of the MIL, leading to a higher conductive solution with increasing the MIL content due to the increase in the ionic charges (cations and anions) into the solution. These ionic charges promote a higher stretching of the jet leading to the formation of thin fibers that appears like spider fibers [29,36]. Further, the incorporation of 10 wt.% results in the broadest fiber distribution comparatively to 5 and 15 wt.% of the MIL. No significant differences are observed in the fiber morphology and fiber diameter between the 10 and 15 wt.% MIL content samples, being the mean fiber diameter within the error 1.12 ± 0.36 µm (Figure 1l). Further, it is also possible to conclude that higher MIL contents do not promote significant changes in the fiber diameter compared to the neat PVDF fibers. It is noteworthy that ILs can be used as porogens in polymer matrices, in which ILs chemically induce a phase separation [37]. The principle of this effect (phase separation) is completely different when compared with common salts, such as sodium chloride (extensively studied to develop porous materials), which are actually removed. In the present case, no phase separation occurs, as demonstrated by the compact fiber structure, the IL being incorporated into the PVDF polymer fibers.

### 3.2. Physical-Chemical Properties

**Figure 2:**
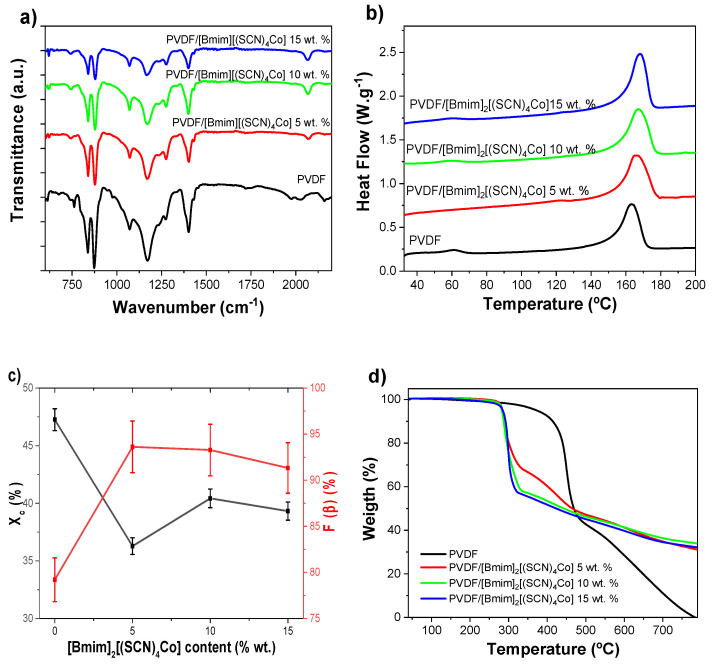
(**a**) ATR-FTIR spectra, (**b**) DSC scans, (**c**) evolution of the *β*-phase content and degree of crystallinity and (**d**) TGA thermograms of the PVDF and PVDF/[Bmim]_2_[(SCN)_4_Co] composites as a function of the MIL content.

The influence of the inclusion of the MIL [Bmim]_2_[(SCN)_4_Co] into the vibration bands, thermal properties and degree of crystallinity of PVDF are shown in Figure 2.

ATR-FTIR was used to evaluate the variations in the polymer phase with the inclusion of the MIL. All spectra show a similar behavior typical of the PVDF/[Bmim]_2_[(SCN)_4_Co] composite fibers containing both crystalline α and electroactive *β*-phases. The characteristic absorption bands of the PVDF polymer are observed in Figure 2a for the *α*-phase at 766, 795 and 855 cm^−1^ and at 840 and 1279 cm^−1^ for the *β*-phase, with no traces of the *γ*-phase [34,38]. The only band related to the MIL is the one observed at ~2060 cm^−1^ in the PVDF/[Bmim]_2_[(SCN)_4_Co] composite fibers, which is related to the anion C-N stretching mode of the MIL and, therefore, its intensity increases with increasing the MIL content [39].

Figure 2b shows the DSC thermograms of the different samples. Similar thermograms are obtained for all samples, characterized by an endothermic peak corresponding to the polymer melting in the range from 150 to 180 °C. It is observed that the value of the melting temperature (*T_m_*) of PVDF is significantly affected by the presence of the MIL. In particular, *T_m_* slightly increases with the concentration of the MIL in the composite fibers from ~164 °C for the PVDF fibers to ~168 °C for the composite fibers containing 15 wt.% of the MIL.

The degree of crystallinity (*X_c_*) was evaluated by using Equation (2). Figure 2c shows the *X_c_* values of the PVDF/[Bmim]_2_[(SCN)_4_Co] composites, which reveal that the addition of the MIL decreases the degree of crystallinity, from 47% for the pristine PVDF to 39% for the sample with 15wt.% content, indicating the strong ion-dipole interactions between the MIL and the PVDF. This phenomenon was already observed in the other PVDF/MIL composites [18,40]. In addition, values of the relative fraction of the *β*-phase material (*F*(*β*)) obtained from FTIR spectra are displayed in Figure 2c. The *β*-phase content of the composites was calculated using Equation (1) and is similar for all samples, with a percentage above 90%. Thus, the processing conditions, i.e., low temperature solvent evaporation and fiber stretching by the applied electric field during the electrospinning process, are the main parameters determining the polymer phase [35], independently of the presence of the MIL.

Figure 2d shows the TGA thermograms of the developed samples. The PVDF displays a single degradation step between ~400 and 500 °C associated to the PVDF chain backbone C-F and C-H bonds scission [41], the total degradation of the pure PVDF occurring at around 800 ºC. Upon the incorporation of 5, 10 and 15 wt.% of the MIL, significant differences between the PVDF samples with and without the MIL are observed. For the PVDF/[Bmim]_2_[(SCN)_4_Co] composites two degradation steps are observed. The first step around 300 °C is attributed to imidazolium cations degradation of the IL [6], and the next steps starting at around 330 °C corresponds to the PVDF decomposition. It is also observed that the shift in the degradation of the PVDF to lower temperatures is proportional to the IL content and can be attributed to the interactions of the IL with the fluorinated polymer matrix (ion-dipole interactions), namely electrostatic interactions between the MIL cations and anions with the negative (CF_2_) and positive (CH_2_) PVDF groups [42]. The TGA thermograms also show a residual mass of ~32% at 800 °C in the samples with different MIL loadings [6,43], probably attributed to the cations and anions residual IL weight.

### 3.3. Magnetic Properties

**Figure 3:**
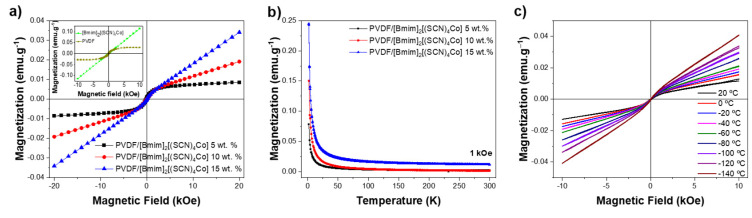
(**a**) Magnetization of the MIL (inset) and composites containing 5, 10 and 15% wt. of [Bmim]_2_[(SCN)_4_Co] at room temperature from −20 kOe to 20 kOe; (**b**) magnetic susceptibility at 1 kOe and (**c**) magnetization of the PVDF/[Bmim]_2_[(SCN)_4_Co] 15 wt.% composite at different temperatures.

Figure 3 shows the magnetization curves of the PVDF, the MIL and the PVDF/[Bmim]_2_[(SCN)_4_Co] hybrid fibers at room temperature, as well as the magnetic behavior of the PVDF/[Bmim]_2_[(SCN)_4_Co] fibers at different temperatures.

The PVDF shows no magnetic response. The magnetic behavior of the composite fibers at room temperature (Figure 3a) shows a paramagnetic behavior, which is stable over time, including after the cytotoxicity assays (Section 3.4), indicating that no leaching of the IL from the fibers occurs. 

The composite fibers present lower magnetization values when compared to the ones observed for magnetic nanoparticles (Fe_3_O_4_ or CoFe_2_O_4_)/polymer electrospun fiber systems [30,31,44]. In addition, the magnetic response also differs with respect to one of magnetic nanoparticle-based systems, which are usually superparamagnetic or ferromagnetic [30,44], unlike the MIL composites paramagnetic response.

The weak ferromagnetism observed in the composite fibers can be attributed to two events: (i) by a contamination with Fe_2_O_3_ during the electrospinning process of the fibers, which can be estimated from the hysteresis loops to be around 90 µg, or (ii) to the ion-dipole electric interaction of the MIL ions, which limits the magnetic movement of the anion [(SCN)_4_Co]^2−^ when imprisoned in the polymeric matrix. This behavior is less noticeable with increasing the MIL content leading to a fully paramagnetic behavior for the composite with 15 wt.% of [Bmim]_2_[(SCN)_4_Co], which is coherent with the paramagnetic nature of the MIL (inset of Figure 3a). Additionally, the slope of the applied field vs the magnetization increases with increasing the MIL content proportionally, indicating that the anion [(SNC)_4_Co]^2−^ spins are weakly interacting and creating an internal magnetic field that follows the same direction as the applied external magnetic field [33]. The ferromagnetic contribution as possible contamination or hindered anion response is compatible with the zero-field cooling that shows a typical paramagnetic behavior and no Verwey transition (Figure 3b). In addition, the hysteresis loops at different temperatures, presented in Figure 3c, show no change on the coercive field.

### 3.4. Cytotoxicity Assays

**Figure 4:**
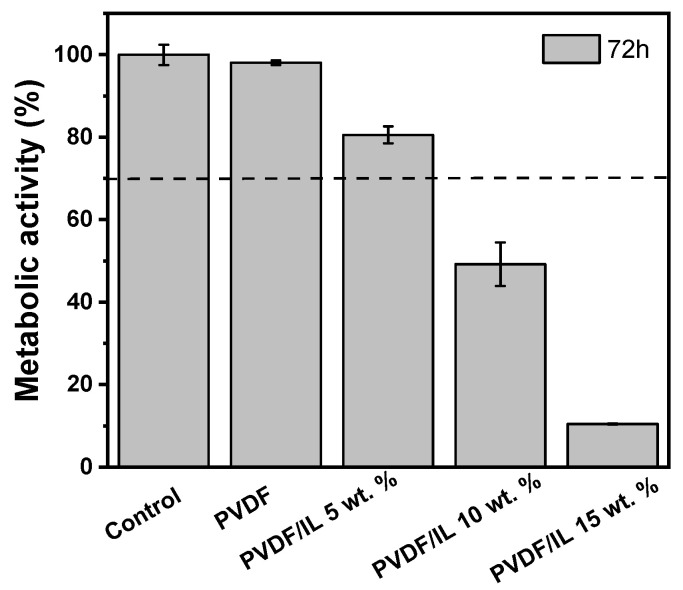
Cytotoxicity assays of L929 cells in contact with the as-prepared extraction media exposed to the PVDF/[Bmim]_2_[(SCN)_4_Co] composites with different [Bmim]_2_[(SCN)_4_Co] contents up to 72 h (relative metabol was presented as the percentage of the negative control (*n* = 5 ± SD)). The bars in the image represents the standard deviation.

The metabolic activity of the PVDF/MIL samples was evaluated by an MTS assay using L929 cell line. According to the ISO standard 10993-5, the samples are considered cytotoxic when a reduction of cell viability by more than 30% occurs [8]. Thus, the cell viability assays allow us to determine the maximum IL concentration that can be incorporate into the PVDF matrix without promoting cell toxicity. As shown in Figure 4, only the 5 wt.% PVDF/[Bmim]_2_[(SCN)_4_Co] composites are not considered cytotoxic. The remaining composites induce a cytotoxic response to the cells, and the metabolic activity decreases with increasing the MIL content, probably as a result of some MIL at the surface of the fibers, which can chemically react with the medium, leading to the formation of some toxic product. Thus, the results indicate that the optimal MIL concentration for biomedical applications is around 5 wt.%.

This may be related to a saturation effect of the maximum amount of the MIL incorporated in the composites. When a higher amount of the MIL is used, not all is incorporated within the polymeric matrix, laying the remaining MIL on the surface of the material.

## 4. Conclusions

The PVDF oriented fiber mats incorporating the MIL [Bmim]_2_[(SCN)_4_Co] up to a maximum of 15 wt.% content was prepared by electrospinning. The addition of the MIL leads to a decrease in the fiber’s diameter when compared to the pristine PVDF fibers, the fiber’s average size ranging from 1.16 ± 0.19 µm for the pristine polymer to 1.12 ± 0.36 µm for the 15wt.% MIL composite. Additionally, the polar *β*-phase content is above ~90% independently of the MIL content and a decrease in the fiber’s degree of crystallinity is observed for the composite fibers, from 47% for the pristine PVDF to 39% for the sample with 15 wt.% content. The melting temperature increases but the thermal degradation of the polymer decreases with the incorporation of the MIL. The magnetization of the PVDF/[Bmim]_2_[(SCN)_4_Co] fibers show a paramagnetic behaviour and is proportional to the MIL content, reaching values up to 0.0348 emu.g^−1^ at 20 kOe for the 15wt.% composite and decreases with decreasing temperature. Finally, the cell viability decreases with increasing the MIL content, showing a non-cytotoxic response for the PVDF and composite fibers with 5 wt.% of MIL, demonstrating their suitability for biomedical applications.

## Supplementary Materials

The following supporting information can be downloaded at: https://www.mdpi.com/article/10.3390/nano12173072/s1, Table S1: Electrospinning processing parameters.
