# Supplementary material for: Electrospun Magnetic Ionic Liquid Based Electroactive Materials for Tissue Engineering Applications
Source: Nanomaterials (Basel). 2022 Sep 4;12(17):3072. doi: 10.3390/nano12173072 (PMC9459776; doi:10.3390/nano12173072)
Supplement: Supplementary file 1 [file nanomaterials-12-03072-s001.zip › nanomaterials-1861274-supplementary.pdf]

nanomaterials

Supplementary Information

Electrospun Magnetic Ionic Liquid Based Electroactive Materials for Tissue Engineering Applications

Liliana . Fernandes [1,2], Rafaela M. Meira [1,2,3], Daniela M. Correia [4,*], Clarisse Ribeiro [1,2], Eduardo Fernandez [5], Carmen R. Tubio [5] and Senentxu Lanceros-Méndez [5,6,*]

[1] Physics Centre of Minho and Porto Universities (CF-UM-UP), University of Minho, 4710-057 Braga, Portugal
[2] LaPMET-Laboratory of Physics for Materials and Emergent Technologies, University of Minho, 4710-057 Braga, Portugal
[3] IB-S-Institute of Science and Innovation for Sustainability, University of Minho, 4710-057 Braga, Portugal
[4] Centre of Chemistry, University of Minho, 4710-057 Braga, Portugal
[5] BCMaterials, Basque Center for Materials, Applications and Nanostructures, UPV/EHU Science Park, 48940 Leioa, Spain
[6] IKERBASQUE, Basque Foundation for Science, 48013 Bilbao, Spain
* Correspondence: dcorreia@quimica.uminho.pt (D.M.C.); senentxu.lanceros@bcmaterials.net (S.L.-M)

Table S1. Electrospinning processing parameters.

**Electrospinning processing parameters**	
Distance between needle and collector	20 cm
Applied voltage	14 kV
Feed rate	0.4 mL h^{-1}
Needle diameter	0.5 mm
Collector velocity	1500 rpm
Relative humidity	40 % - 60 %
Temperature	25 ºC
